# Lactobacillus GG restoration of the gliadin induced epithelial barrier disruption: the role of cellular polyamines

**DOI:** 10.1186/1471-2180-14-19

**Published:** 2014-01-31

**Authors:** Antonella Orlando, Michele Linsalata, Maria Notarnicola, Valeria Tutino, Francesco Russo

**Affiliations:** 1 Laboratory of Nutritional Pathophysiology, National Institute for Digestive Diseases I.R.C.C.S. “Saverio de Bellis”, via Turi 27, I-70013 Castellana Grotte, BA, Italy; 2 Laboratory of Nutritional Biochemistry, National Institute for Digestive Diseases I.R.C.C.S. “Saverio de Bellis”, via Turi 27, I-70013 Castellana Grotte, BA, Italy

**Keywords:** Caco-2 cells, Celiac disease, Gliadin, Intestinal barrier function, *Lactobacillus rhamnosus* GG, Paracellular permeability, Polyamines, Tight junction proteins

## Abstract

**Background:**

Celiac disease is characterized by enhanced intestinal paracellular permeability due to alterations of function and expression of tight junction (TJ) proteins including ZO-1, Claudin-1 and Occludin. Polyamines are pivotal in the control of intestinal barrier function and are also involved in the regulation of intercellular junction proteins. Different probiotic strains may inhibit gliadin-induced toxic effects and the *Lactobacillus rhamnosus* GG (L.GG) is effective in the prevention and treatment of gastrointestinal diseases. Aims of the study were to establish in epithelial Caco-2 cells whether i) gliadin affects paracellular permeability and polyamine profile; ii) co-administration of viable L.GG, heat-killed L.GG (L.GG-HK) or its conditioned medium (L.GG-CM) preserves the intestinal epithelial barrier integrity. Additionally, the effects of L.GG on TJ protein expression were tested in presence or absence of polyamines.

**Results:**

Administration of gliadin (1 mg/ml) to Caco-2 cells for 6 h caused a significant alteration of paracellular permeability as demonstrated by the rapid decrease in transepithelial resistance with a concomitant zonulin release. These events were followed by a significant increase in lactulose paracellular transport and a slight lowering in ZO-1 and Occludin expression without affecting Claudin-1. Besides, the single and total polyamine content increased significantly. The co-administration of viable L.GG (10^8^ CFU/ml), L.GG-HK and L.GG-CM with gliadin significantly restored barrier function as demonstrated by transepithelial resistance, lactulose flux and zonulin release. Viable L.GG and L.GG-HK, but not L.GG-CM, led to a significant reduction in the single and total polyamine levels. Additionally, only the co-administration of viable L.GG with gliadin significantly increased ZO-1, Claudin-1 and Occludin gene expression compared to control cells. When Caco-2 cells treated with viable L.GG and gliadin were deprived in the polyamine content by α-Difluoromethylornithine, the expression of TJ protein mRNAs was not significantly different from that in controls or cells treated with gliadin alone.

**Conclusions:**

Gliadin modifies the intestinal paracellular permeability and significantly increases the polyamine content in Caco-2 cells. Concomitant administration of L.GG is able to counteract these effects. Interestingly, the presence of cellular polyamines is necessary for this probiotic to exert its capability in restoring paracellular permeability by affecting the expression of different TJ proteins.

## Background

Celiac disease (CD) is a chronic inflammatory disease in the small intestine of genetically predisposed individuals triggered by the gluten fraction of wheat, rich in glutamine and proline, or the homologous proteins from barley and rye. The major part of toxic components is contained in gliadin, the alcohol-soluble fraction of gluten. In humans, the undigested molecules of gliadin are resistant to degradation by gastric, pancreatic, and intestinal brush-border membrane proteases and thus remain in the intestinal lumen after gluten ingestion [1].

CD is characterized by enhanced paracellular permeability and an impairment in the integrity of the intestinal barrier [2] that allows the interactions of gluten peptides with antigen-presenting cells in the lamina propria. Gliadin is rich in glutamine and the presence of numerous glutamine acceptor proteins in the extracellular matrix could be responsible for the formation of cross-links between gliadin and matrix proteins. In turn, this gliadin immobilization to extracellular matrix proteins could provide a long-term availability of toxic gliadin fractions in the mucosa [3]. However, there is still much debate about the possible interactions of gliadin (and/or its peptide derivatives) with intestinal epithelia and the mechanism(s) through which it crosses the epithelial barrier to reach the submucosa [4].

Integrity of the intestinal barrier depends on a complex of proteins composing different intercellular junctions, including tight junctions (TJs) and adherens junctions [5]. Major transmembrane and cytosolic TJ proteins in the mammalian epithelium include Zonula Occludens ZO-1 and ZO-2, Occludin and Claudins. These proteins are thought to constitute the backbone of TJ strands and to modulate some functions of TJs, respectively [6]. ZO-1 and ZO-2 are the cytoplasmic faces of TJs and directly bind to the COOH terminus of intracellular domain of Occludin. The interaction between Occludin and ZO-1 or ZO-2 protein is crucial for maintaining normal structure of the TJs and epithelial barrier function [6]. Occludin is a 65-kDa integral plasma-membrane protein. Its expression is markedly decreased in intestinal permeability (IP) disorders, including Crohn’s disease, ulcerative colitis and CD, suggesting that the lowering in Occludin expression may play a part in the increase in IP [7]. The Claudin family of TJ proteins regulates the epithelial paracellular permeability. Claudins are 20- to 27-kDa proteins containing 2 extracellular loops with variably charge aminoacid residues among family members and short intracellular tails [8]. In intestinal epithelial cells, Claudin-1 expression is associated with enhancement of epithelial barrier function [9] and it is found to be decreased in both intestinal and extraintestinal diseases [10].

Among the several substances involved in the IP control, polyamines play a crucial role. These polycationic compounds are ubiquitous short-chain aliphatic amines present in all the eukaryotic cells studied and regulate cell proliferation and differentiation [11]. Polyamines are also involved in the expression and functions of intercellular junction proteins, as well as in maintenance of intestinal epithelial integrity [12]. With their positive charges, polyamines can form bridges between distant negative charges, resulting in unique effects on permeability. The action of polyamines in modulating IP to different-sized markers generally seems to depend on their concentration [13]. Spermidine appears to enhance mucosal permeability to macromolecules at lower concentration (1 mM), as compared to putrescine (10 mM).

The protective effect of polyamines on the *in vitro* toxicity of gliadin peptides has been related to their effect on the functions of intestinal brush border or intracellular membranes involved in the handling of gliadin and initial studies suggested that amines could act as transglutaminase amino donor substrates in the intestinal metabolism of gliadin peptides [14]. However, little is still known about the direct action of gliadin on the levels of polyamines in *in vitro* cell conditions.

At present, a strict, lifelong gluten-free diet (GFD) is the only CD treatment. Therefore, alternative strategies for treating CD are being hypothesized including agents that are able to counteract the gluten induced damage on epithelial mucosa.

Probiotic bacteria have been shown to preserve the intestinal barrier promoting its integrity both *in vitro* and *in vivo*[15,16]. Besides, different probiotic strains may show promising abilities in inhibiting gliadin-induced toxic effects [17] and a particular lactobacillus strain, the *Lactobacillus rhamnosus* GG (ATCC 53103) (L.GG), has shown properties in the prevention and treatment of different gastrointestinal diseases [18]. L.GG is one of the clinically best-studied probiotic organisms and displays very good *in vitro* adherence to epithelial cells and mucus. In previous studies by our group this strain, when tested as both viable and heat inactivated bacteria as well as homogenate and cytoplasm extracts, has also been demonstrated *in vitro* to significantly affect cell proliferation and polyamine metabolism [19,20].

On these bases, the present *in vitro* study aimed at answering to the following questions: a) does gliadin affect the paracellular permeability and polyamine profile? b) does concomitant administration of viable L.GG, heat-killed L.GG or its conditioned medium preserve the intestinal epithelial barrier, after disruption with gliadin? c) what are their effects on the TJ protein expression? The role of cellular polyamines as a requisite for L.GG action on the expression of TJ proteins was also investigated.

As *in vitro* model of CD the Caco-2 cell line was used. This line is formed by intestinal epithelial cells obtained from human colon adenocarcinoma, that, before confluence, mimics the physiological enterocytes, and provides an important and widely used tool for studying and obtaining greater insight into the molecular and cellular mechanisms of CD alterations in epithelial cells [21].

## Methods

### Cell culture conditions

Human colon adenocarcinoma-derived Caco-2 cells were obtained from the Interlab Cell Line Collection (IST, Genoa, Italy). Cells were routinely cultured in RPMI-1640 medium supplemented with 10% fetal bovine serum (FBS), 2 mM glutamine, 100 U/ml penicillin, 100 μg/ml streptomycin, in a monolayer culture, and incubated at 37°C in a humidified atmosphere containing 5% CO_2_ in air. At confluence, the grown cells were harvested by means of trypsinization and serially subcultured with a 1:4 split ratio. All cell culture components were purchased from Sigma-Aldrich (Milan, Italy).

### Bacterial strain

As probiotic, the *Lactobacillus rhamnosus* ATCC 53103 (commercially named *Lactobacillus* GG, L.GG, obtained from the American Type Culture Collection ATCC, Manassas, VA USA) was tested in our set of experiments.

L.GG was cultured at 37°C for 24 h under anaerobic conditions in Man-Rogosa-Sharpe (MRS) broth; the incubate was centrifuged (300 × g for 10 min) at room temperature and the precipitate was collected and washed twice with phosphate buffered saline (PBS) at pH 7.4. The bacteria were then re-suspended in RPMI-1640 medium in order to give a bacterial concentration of 10^8^ CFU/ml (as determined by colony counts). Heat-treatment of L.GG was performed by heating at 95°C for 1 h. Bacterial conditioned medium (CM) was collected by centrifugating the incubate at 300 × g for 10 min. The supernatant (conditioned medium) was filtered through a 0.22 μm low-protein-binding filter (Millex; Millipore, Bedford, MA) to sterilize and remove all bacterial cells. Aliquots of L.GG-CM were stored in sterile microcentrifuge tubes at −20°C until use. Caco-2 cells were treated with LGG-CM as a 10% volume of the total incubation cell medium.

### Gliadin and L.GG treatments

Caco-2 cells (25th-30th passage) were seeded at a density of 2 × 10^5^ cells/5 ml of supplemented RPMI-1640 in 60 mm tissue culture dishes (Corning Costar Co., Milan, Italy). After 24 h, to allow for attachment, the medium was removed and RPMI-1640 supplemented with 10% FBS and 2 mM glutamine, containing viable L.GG (10^8^ CFU/ml), L.GG-heat killed (L.GG-HK), L.GG-CM were added to cells for 6 h. A concomitant set of experiments was conducted by administering for the same time 1 mg/ml wheat gliadin (Sigma-Aldrich) alone or in combination with viable L.GG, L.GG-HK and L.GG-CM. Triplicate cultures were set up for each treatment and for the control, and each experiment was repeated 3 times.

In the experiments investigating the transepithelial resistance (TER), zonulin release and lactulose flux after the above cited treatments, Caco-2 cells were plated onto Millicell Culture inserts (Millipore Corporate, Billerica, MA, USA); 2 ml of supplemented RPMI was added to the mucosal (apical) side and 3 ml of the same medium was added to the serosal (basolateral) side. Cells were incubated at 37°C in an atmosphere of 95% air and 5% CO_2_ and grown until confluence (average 10–15 days post-seeding). Then, the monolayer was washed with PBS twice and incubated with RPMI supplemented as above but without antibiotics. Replicates of Caco-2 monolayers were incubated at increasing time intervals (0–30 min - 60 min- 90 min - 3 h - 6 h) after undergoing the above described gliadin and L.GG treatments. The preparations were added to the mucosal (apical) side of the Caco-2 monolayers.

### Transepithelial resistance measurements

The resistance of the cell monolayer was measured using a Millicell-ERS volt-ohm meter (Millipore Corporate). Caco-2 cells were regarded as confluent when TER exceeded 600 ohms/cm^2^[17]. Confluent monolayers were washed twice with PBS and incubated overnight in RPMI medium supplemented with 10% FBS and 2 mM glutamine but without antibiotics prior to gliadin and L.GG treatments. After cell exposure to bacteria and/or gliadin, TER was measured immediately after changing the media as well as after 30 min, 60 min, 90 min, 3 h, and 6 h.

### Measurement of lactulose flux from the apical to basolateral side of Caco-2 monolayers

Lactulose, a probe used to check paracellular permeability, was added at 40 mM/ml final concentration to the apical side of all monolayers at time 0. Samples were collected from the basolateral side at increasing time intervals (ranging from 30 min to 6 h) after gliadin and L.GG treatments. Lactulose concentration was measured by high performance anion exchange chromatography (HPAEC) [22]. After deproteination with acetonitrile 1:1 v/v, samples were centrifuged at 4000 rpm for 10 min, the supernatant collected, filtered through a 0.22 mm membrane (Millipore, Bedford, Mass., USA), and diluted with water 1 to 10 (basolateral samples) or 1 to 100 (apical samples). HPAEC coupled with pulsed amperometric detection (HPAEC-PAD) was performed on a Dionex Model ICS-5000 with a gold working electrode and a 25 μl peek sample loop (Dionex Corp., Sunnyvale, CA, USA). Carbohydrate separation was carried out by a Carbopac PA-10 pellicular anion-exchange resin connected to a Carbopac PA-10 guard column at 30°C.

### Zonulin determination

Zonulin content from cell culture media was quantified at increasing time intervals (ranging from 30 min to 6 h) after gliadin and L.GG treatments, using the zonulin enzyme-linked immunosorbent assay (Elisa) kit (Immunodiagnostik, Bensheim, Germany) [23].

### Polyamine analysis

For the evaluation of polyamine levels after gliadin and L.GG treatments for 6 h, each cell culture pellet was homogenized in 700 μl of 0.9% sodium chloride mixed with 10 μl (200 nmol/ml) of the internal standard 1,10-diaminodecane (1,10-DAD). An aliquot of the homogenate was used to measure the total protein content. Then, to precipitate proteins, 50 μl of perchloride acid (PCA) 3 M were added to the homogenate. After 30 min of incubation in ice, the homogenate was centrifuged for 15 min at 7000 × g. The supernatant was filtered (Millex-HV13 pore size 0.45 μm, Millipore, Bedford, MA, USA) and lyophilized. The residue was dissolved in 300 μl of HCL (0.1 N). Dansylation and the extraction of dansyl-polyamine derivatives were performed as previously described [24]. After extraction, aliquots of 200 μl were injected into a high-performance liquid chromatography system (UltiMate 3000, Dionex Corp., Sunnyvale, CA, USA) equipped with a reverse-phase column (Sunfire C18, 4.6 × 100 mm, 3.5 μm particle size, Waters, Milford, MA, USA). Polyamines were eluted with a linear gradient ranging from acetonitrile-water (50:50, v:v) to acetonitrile (100%) for 30 min. The flow was 0.5-1.0 ml/min from 0 to 12 min and then set at a constant rate (1.0 ml/min) until the 30th min. The fluorescent intensity was monitored by a fluorescence detector (UltiMate 3000 RS, Dionex Corp., Sunnyvale, CA, USA) with excitation at 320 nm and emission at 512 nm. Polyamine levels were expressed as concentration values in nmol/mg of protein.

### ZO-1, claudin-1 and occludin expression

The effects of gliadin and L.GG treatments for 6 h and 24 h on ZO-1, Claudin-1 and Occludin mRNA and protein levels in Caco-2 cells were evaluated using the quantitative PCR (qPCR) method with SYBR1 green dye and Western Blot analysis, respectively. Besides, to investigate whether the potential changes in TJ expression following to the combined administration of viable L.GG with gliadin could be related to the polyamine content, the cells were cultured with α-Difluoromethylornithine (DFMO) 5 mM for 4 days before undergoing the same treatment for 6 h. DFMO is a specific inhibitor of polyamine synthesis and, as reported in literature, at a concentration of 5 mM, it is able to completely deplete putrescine within 48 h and to totally deplete spermidine and reduce by 60% spermine within 4 days [25].

Cells were washed twice in PBS and then trypsinized and centrifuged at 280 × g. The cell pellets were resuspended in 0.3 ml of pure distilled water and used for RNA extraction. Total cell RNA was extracted using Tri-Reagent (Mol. Res. Center Inc., Cincinnati, Ohio, USA), following the manufacture’s instruction. About 2 μg total cell RNA, extracted from both the control and treated cells, was used for cDNA synthesis. Reverse transcription (RT) was carried out in 20 μl of the final volume at 41°C for 60 min, using 30 pmol antisense primer (Table 1) for analyses of the ZO-1, Claudin-1, Occludin and the β-actin gene [26]. The β-actin gene was utilized as an internal control and was chosen as a reference gene because it is a housekeeping gene. Real-time PCRs were performed in 25 μl of final volume containing 2 μl of cDNA, master mix with SYBR Green (iQ SYBR Green Supermix Bio-Rad, Milan, Italy) and sense and antisense primers for the ZO-1, Claudin-1, Occludin and the β-actin gene (Table 1).

**Table 1:** Sequences of amplification primers

**Gene**		**Primer**
ZO-1	Sense	5′- ATCCCTCAAGGAGCCATTC-3′
	Antisense	5′- CACTTGTTTTGCCAGGTTTTA-3′
Claudin-1	Sense	5′- AAGTGCTTGGAAGACGATGA-3′
	Antisense	5′- CTTGGTGTTGGGTAAGAGGTT-3′
Occludin	Sense	5′-CCAATGTCGAGGAGTGGG-3′
	Antisense	5′-CGCTGCTGTAACGAGGCT-3′
β-actin	Sense	5′-AAAGACCTGTACGCCAACACAGTGCTGTCTGG-3′
	Antisense	5′-CGTCATACTCCTGCTTGCT GATCCACATCTGC-3

Real-time PCRs were carried out in a CFX96 Real-Time PCR Detection System (Bio-Rad Laboratories, Inc.) using the following protocol: 45 cycles at 95°C for 3 min, 95°C for 10 s, 55°C for 30 s followed by a melting curve step at 65 – 95°C with a heating rate of 0.5°C per cycle for 80 cycles. The PCR products were quantified by external calibration curves, one for each tested gene, obtained with serial dilutions of known copy number of molecules (10^2^-10^7^ molecules). All expression data were normalized by dividing the target amount by the amount of β-actin used as internal control for each sample. The specificity of the PCR product was confirmed by gel electrophoresis.

As Western Blot concerns, Caco-2 cells were collected and lysed on ice in RIPA buffer (Pierce Ripa buffer, Thermo Scientific, Rockford, IL, USA). After homogenization and centrifugation at 14000 rpm for 15 min at 4°C, protein concentration was measured by a standard Bradford assay (Bio-Rad Laboratories, Milan, Italy). Aliquots of 50 μg of total proteins were separated in 4-12% pre-cast polyacrylamide gels (Invitrogen, Life Technologies, OR, USA) and transferred onto a PVDF membrane (Bio-Rad Laboratories, Milan, Italy) with Transblot Turbo (Bio-Rad Laboratories). ZO-1, Claudin-1, Occludin and β-actin protein expressions were evaluated by 1:500 diluted ZO-1 (H-300), Claudin-1 (D-4), Occludin (N-19) and β-actin antibody, respectively (Santa Cruz Biotechnology, Santa Cruz, CA, USA). After overnight incubation, the membranes were further incubated with a horseradish peroxidase-conjugated goat secondary antibody (Bio-Rad Laboratories). The proteins were detected by chemiluminescence (ECL, Thermo Scientific, Rockford, IL, USA) and the densitometric analysis of each protein-related signal was obtained using the Molecular Imager Chemidoc™ (Bio-Rad Laboratories) and normalized against β-actin expression.

### Statistical analysis

Due to the non-normal distribution of the data, non-parametric tests were performed. Data were analyzed by Kruskal-Wallis analysis of variance and Dunn’s Multiple Comparison Test. In order to evaluate the release of zonulin during the time of observation, the area under the curve (AUC) was calculated. All data are expressed as mean and SEM. Differences were considered significant at P < 0.05. A specific software package (SigmaStat for Windows version 3.00 SPSS Inc. San Jose, CA, USA) was used.

## Results

### Effects of gliadin and L.GG treatments on Caco-2 monolayer barrier function (TER and lactulose flux)

TER measurements were determined after the addition of viable L.GG, L.GG-HK and L.GG-CM to polarized monolayers of Caco-2 cells seeded on Transwell filter inserts. TER was measured before the addition of bacteria, at time zero (immediately after the bacteria administration) and then at various time intervals ranging from 30 min to 6 h. A slight and not significant increase in TER was observed after 90 min from the addition of viable L.GG, as well as L.GG-HK and L.GG-CM to Caco-2 cells compared to control cells (data not shown).

**Figure 1:**
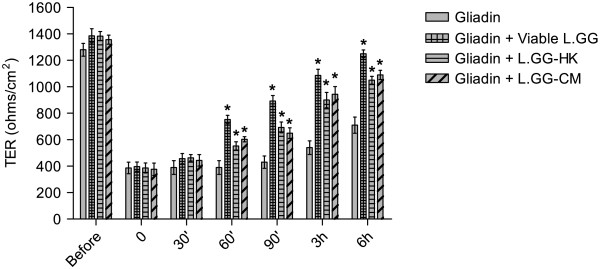
**Effects of supplementation of viable L.GG (10**^**8**^ **CFU/ml), L.GG-HK and L.GG-CM on gliadin-induced (1 mg/ml) TER decrease.** All data represent the results of three different experiments (mean ± SEM). For each time of treatment, data were analyzed by Kruskal-Wallis analysis of variance and Dunn’s Multiple Comparison Test. (*) P < 0.05 compared to gliadin treated cells.

Addition of gliadin to the mucosal side of Caco-2 monolayers led to an immediate lowering in TER (Figure 1). The TER of gliadin-treated Caco-2 cells immediately after the gliadin administration (time zero) decreased to 30% of TER measured before treatment and started to recover after 90 min of incubation. The co-administration of viable L.GG, L.GG-HK and L.GG-CM with gliadin had a significant (P < 0.05) reversible effect on the recovery of TER starting 60 min post-incubation compared to gliadin-treated cells. After 6 h, the reversion of TER of viable L.GG, L.GG-HK and L.GG-CM to gliadin-treated cells reached 90%, 76% and 80% of their initial values before the addition of gliadin.

To confirm that TER reduction involved the opening of intercellular TJs, the mucosal to serosal transport of the paracellular marker lactulose was also monitored. No effect on lactulose flux was observed after 90 min from the addition of viable L.GG, as well as L.GG-HK and L.GG-CM to Caco-2 cells compared to control cells (data not shown). By opposite, in monolayers treated with gliadin, a significant increase (P < 0.05) in serosal lactulose (0.077 ± 0.04 μg/ml) was observed 90 min after gliadin exposure compared to untreated monolayers (0.025 ± 0.02 μg/ml). The co-administration of viable L.GG, L.GG-HK and L.GG-CM antagonized the increased paracellular lactulose transport due to gliadin treatment (viable L.GG: 0.03 ± 0.02 μg/ml; L.GG-HK: 0.039 ± 0.01 μg/ml; L.GG-CM: 0.04 ± 0.01 μg/ml).

### Effects of gliadin and L.GG treatments on zonulin release

**Figure 2:**
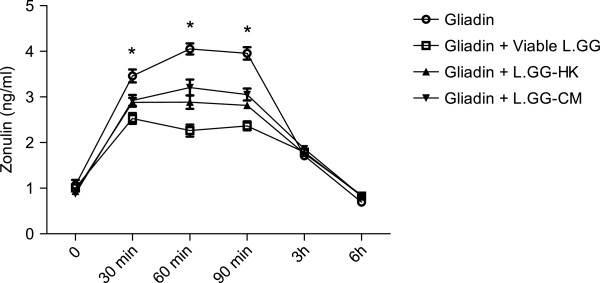
**Zonulin release in Caco-2 monolayers exposed to gliadin (1 mg/ml) alone or in combination with viable L.GG (10**^**8**^ **CFU/ml), heat killed L.GG (L.GG-HK) and L.GG conditioned medium (L.GG-CM).** All data represent the results of three different experiments (mean ± SEM). For each time of treatment, data were analyzed by Kruskal-Wallis analysis of variance and Dunn’s Multiple Comparison Test. (*) P < 0.05 gliadin *vs.* gliadin + Viable L.GG.

Viable L.GG, L.GG-HK and L.GG-CM were administered to Caco-2 cells at different times (ranging from 0 to 6 h). No effect on zonulin release was observed (data not shown). The incubation of Caco-2 cells with gliadin led to a significant (P < 0.05) luminal secretion of zonulin starting from 30 min post-incubation (Figure 2). Zonulin release reached baseline values after 6 h of exposure. The co-administration of viable L.GG, L.GG-HK and L.GG-CM with gliadin counteracted the zonulin release induced by gliadin. The differences in the zonulin levels were significant between cells treated with gliadin and cell treated with gliadin and viable L.GG at 30 min, 60 min and 90 min (P < 0.05) (Figure 2).

In order to calculate the differences in the zonulin release over the time of exposure to gliadin alone or in combination with viable L.GG, L.GG-HK and L.GG-CM at different times (ranging from 0 min to 6 h), the AUCs of zonulin were calculated. The AUC value was higher in the gliadin-treated Caco-2 cells (14.06 ± 0.54) compared to those in cells treated with gliadin and viable L.GG (9.86 ± 0.28), gliadin and L.GG-HK (11.20 ± 0.33) and gliadin and L.GG-CM (11.93 ± 0.45). The difference was significant (P = 0.02) between Caco-2 cells treated with gliadin alone and cells treated with gliadin and viable L.GG.

### Effects of gliadin and L.GG treatments on the polyamine profile

The effects of viable L.GG, L.GG-HK and L.GG-CM on the polyamine profile of Caco-2 cell line were studied (Table 2). The administration of viable L.GG and L.GG-HK, but not L.GG-CM, led to a decrease of the single and total polyamine contents. The decrease was significant (P < 0.05) for spermidine, spermine and the total polyamine content compared to untreated control cells.

**Table 2:** **Polyamine profile in Caco-2 cells after 6 h of exposure to viable L.GG (10**^**8**^ **CFU/ml), L.GG-HK and L.GG-CM, alone or in combination with gliadin (1 mg/ml)**

	**Control**	**Viable L.GG**	**L.GG-HK**	**L.GG-CM**	**Gliadin**	**Gliadin + Viable L.GG**	**Gliadin + L.GG-HK**	**Gliadin + L.GG-CM**
***Putrescine***	0.15 ± 0.1^a^	0.12 ± 0.1^a^	0.1 ± 0.2^a^	0.12 ± 0.1^a^	0.2 ± 0.005^a^	0.2 ± 0.008^a^	0.16 ± 0.005^a^	0.2 ± 0.01^a^
***Spermidine***	6.9 ± 0.08^a^	3.3 ± 0.1^c^	3.8 ± 0.2^c^	6.8 ± 0.09^a^	9.3 ± 0.05^b^	6.0 ± 0.06^a^	7.1 ± 0.05^a^	8.2 ± 0.2^ab^
***Spermine***	7.8 ± 0.05^a^	4.3 ± 0.04^c^	5.3 ± 0.5^c^	7.5 ± 0.05^a^	11.1 ± 0.3^b^	4.3 ± 0.1^c^	8.9 ± 0.03^a^	11.3 ± 0.09 ^ab^
***Total polyamines***	14.3 ± 0.3^a^	7.9 ± 0.5^c^	9.1 ± 0.6^c^	14.4 ± 0.5^a^	20.9 ± 0.8^b^	10.3 ± 0.4^c^	15.9 ± 0.3^a^	20.01 ± 0.5^b^

All data represent the results of three different experiments (mean ± SEM). For each treatment mean values not sharing a common superscript differ significantly (P < 0.05, Kruskal-Wallis analysis of variance and Dunn’s Multiple Comparison Test). Polyamines are in nmol/mg protein.

The administration of gliadin to Caco-2 cells led to a significant increase (P < 0.05) in the spermidine (+35%), spermine (+42%) and total polyamine content (+46%) in comparison with untreated control cells.

The supplementation of viable L.GG and L.GG-HK, but not L.GG-CM, on gliadin treated cells counteracted significantly (P < 0.05) the effects of gliadin on the polyamine profile. In particular, the contents in spermidine and spermine decreased by 35.5% and 61.3%, respectively for viable L.GG. Overall, the percentage of reduction in the total polyamine content was by 50.7%. As concerns cells treated with gliadin and L.GG-HK, the reduction in spermidine and spermine content was equal to 23.6% and 19.8%, respectively. The total polyamine content was reduced by 23.9%.

### Effects of gliadin and L.GG treatments on ZO-1, Claudin-1 and Occludin expression

To establish whether the changes in paracellular permeability on Caco-2 monolayers following gliadin and L.GG treatments were associated with modifications in ZO-1, Claudin-1 and Occludin expression, mRNA and protein levels of the three proteins were quantified by qPCR and Western Blot analysis, respectively.

**Figure 3:**
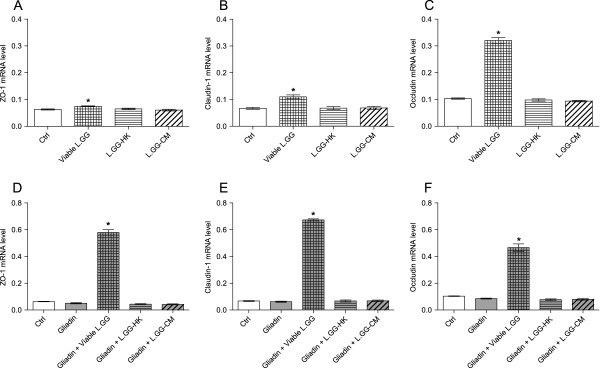
**ZO-1, Claudin-1 and Occludin mRNA levels in Caco-2 monolayers after 6 h of exposure to different probiotic and gliadin treatments.** Panels **A**, **B**, and **C** report ZO-1, Claudin-1 and Occludin mRNA levels in Caco-2 monolayers after 6 h of exposure to viable L.GG (10^8^ CFU/ml), heat killed L.GG (L.GG-HK) and L.GG conditioned medium (L.GG-CM). Data were analyzed by Kruskal-Wallis analysis of variance and Dunn’s Multiple Comparison Test. (*) P < 0.05 compared to control cells. Panels **D**, **E** and **F** report ZO-1, Claudin-1 and Occludin mRNA levels in Caco-2 monolayers after 6 h of exposure to gliadin (1 mg/ml) alone or in combination with viable L.GG, L.GG-HK and L.GG-CM. Data were analyzed by Kruskal-Wallis analysis of variance and Dunn’s Multiple Comparison Test. (*) P < 0.05 compared to gliadin treated cells. All data represent the results of three different experiments (mean ± SEM).

When Caco-2 cells were exposed to viable L.GG, L.GG-HK and L.GG-CM for 6 h, a significant (P < 0.05) increase in the ZO-1, Claudin-1 and Occludin mRNA levels compared to control cells was observed only after viable bacteria treatment (Figure 3, panels A, B, and C).

The administration of gliadin did exert a slight and not significant down-regulatory effect on ZO-1 (−20.6%) and Occludin (−17.5%) expression, without affecting Claudin-1 one. By opposite, only the administration of viable L.GG in combination with gliadin caused a significant (P < 0.05) increase in the mRNA levels of all the tested proteins. In particular, ZO-1 and Claudin-1 increased more than tenfold and Occludin more than fourfold compared to gliadin-treated cells (Figure 3, panels D, E, and F). L.GG-HK and L.GG-CM in combination with gliadin did not exert any significant effect on mRNA levels of the three proteins.

This finding was a little contradictory. It would be expected to see differences also in the TJ mRNA levels of the gliadin treated cells compared to controls. Therefore, ZO-1, Claudin-1 and Occludin expressions were evaluated in function of the time, following 24 h of exposure. ZO-1 and Claudin-1 mRNA levels were significantly (P < 0.05) affected by exposure to gliadin compared to untreated control cells. In particular ZO-1 expression decreased by 25% (0.80 ± 0.04 *vs.* 0.60 ± 0.01) while Claudin-1 decreased by 80% (0.05 ± 0.02 *vs.* 0.01 ± 0.01). Occludin expression remained unchanged (0.04 ± 0.02 *vs.* 0.035 ± 0.02). These results suggest that gliadin may be involved in the regulation of the TJ expression in a time dependent fashion. The administration of viable L.GG in combination with gliadin continued to significantly (P < 0.05) increase the mRNA levels of Claudin-1 (2.27 ± 0.06 *vs.* 0.037 ± 0.01) and Occludin (1.3 ± 0.02 *vs.* 0.12 ± 0.02) while exerting a slight and not significant decrease on ZO-1 expression (0.79 ± 0.02 *vs.* 1.04 ± 0.04) compared to gliadin treated cells.

**Figure 4:**
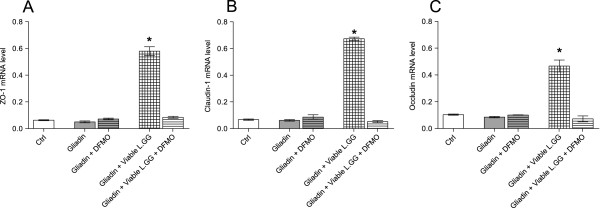
**ZO-1, Claudin-1 and Occludin mRNA levels in Caco-2 monolayers after 6 h of exposure to gliadin (1 mg/ml) alone or in combination with viable L.GG (10**^**8**^ **CFU/ml), in presence or absence of polyamines following administration of α-Difluoromethylornithine (DFMO).** All data represent the results of three different experiments (mean ± SEM). **A**. ZO-1 mRNA levels; **B**. Claudin-1 mRNA levels; **C**. Occludin mRNA levels. Data were analyzed by Kruskal-Wallis analysis of variance and Dunn’s Multiple Comparison Test. (*) P < 0.05 compared to gliadin treated cells.

Given that only viable L.GG was effective in modulating TJ expression, alone or in combination with gliadin, we investigated whether the presence of cellular polyamines could affect the action of viable L.GG on TJ protein expression. Therefore, a subsequent set of experiments was conducted also in absence of polyamines by treating Caco-2 cells with DFMO for 6 h. The addition of gliadin to cells did not significantly influence the expression of all the proteins. Interestingly, also the supplementation of viable L.GG to gliadin did not produce consequences on the mRNA levels of ZO-1, Claudin-1 and Occludin and this evidence suggests the need of polyamines by this probiotic to exert its actions on TJ protein expression (Figure 4, panels A, B, and C).

**Figure 5:**
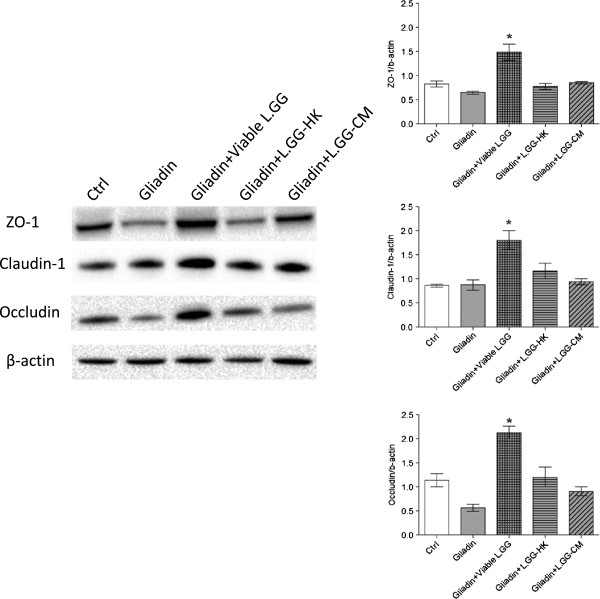
**Western Blot analysis of ZO-1, Claudin-1 and Occludin (using their specific antibodies as specified in Methods) in Caco-2 monolayers after 6 h of exposure to gliadin (1 mg/ml) alone or in combination with viable L.GG (10**^**8**^ **CFU/ml), heat killed L.GG (L.GG-HK) and L.GG conditioned medium (L.GG-CM).** Immunoreactive bands were quantified using Quantity One programme. The diagrams show quantification of the intensity of bands, calibrated to the intensity of the β-actin bands. All data represent the results of three different experiments (mean ± SEM). Data were analyzed by Kruskal-Wallis analysis of variance and Dunn’s Multiple Comparison Test. (*) P < 0.05 compared to gliadin treated cells.

Overall, Western Blot analysis confirmed the results obtained by qPCR at 6 h and 24 h. In particular, Figure 5 reports the results obtained at 6 h. The protein levels of ZO-1 and Occludin in Caco-2 cells decreased not significantly after treatment with gliadin alone compared to control cells. Claudin-1 was not affected in its levels. Besides, the co-administration of gliadin with viable L.GG, but not with L.GG-HK and L.GG-CM, led to a significant increase (P < 0.05) in the levels of ZO-1 (twofold), Claudin-1 (twofold) and Occludin (fourfold) compared to gliadin-treated cells.

## Discussion

In physiological conditions, intestinal epithelium is impermeable to macromolecules, but in CD patients the gliadin fraction of wheat gluten represents the environmental factor responsible for the alterations in the junctional structures between epithelial cells leading to compromised permeability [4].

In our *in vitro* conditions, administration of gliadin to Caco-2 cells caused an increase in paracellular permeability as demonstrated by the dramatic decrease in TER immediately after the exposure, with a concomitant release of zonulin. These events were followed at 90 min by a significant rising in the lactulose paracellular transport. Overall, the process was rapid. After 6 h from exposure, the release of zonulin was similar to baseline values. It is now accepted that one of the immediate consequences of gluten exposure is the increased paracellular permeability, occurring within 36 h [27] and our observations along with data in literature from *in vivo* studies, support that this is an early event rather than a consequence of chronic intestinal inflammation [22].

CD patients show structural alterations at TJs that are made up of transmembrane proteins such as Occludins, and Claudins with intra-cellular connections to the Zonulins, which are members of the ZO family. These, in turn, are anchored to the cell’s actinomyosin cytoskeleton and the result is a structure that not only provides the epithelium with a barrier function but also, by rapid assembly and disassembly, changes its permeability upon different stimuli [28]. In our study, ZO-1, Claudin-1 and Occludin expression was assessed to test their involvement in modifications of paracellular permeability of Caco-2 cells. When these cells were exposed to gliadin, a time dependent effect on TJs expression was observed. After 6 h of gliadin exposure, a slight and not significant decrease in ZO-1 and Occludin expression occurred without affecting Claudin-1. By prolonging the time of exposure up to 24 h, ZO-1 and Claudin-1 expressions decreased significantly while Occludin expression remained unchanged. This evidence let us hypothesize that the continuous exposure to a toxic agent such as gliadin could bring cells to a rearrangement in TJ architecture, beyond the immediate disruption of the epithelial barrier. Few data are available on this item. Previously, Sander et al. [29] reported a fast disruption of intestinal barrier function in Caco-2 cells (after 4 h of exposure to gliadin peptic-tryptic digest) that markedly involved Occludin, ZO-1 and E-cadherin. In our study, the events were not so rapid even if, in agreement with these authors, we also found that permeability, as measured by TER, increased immediately after gliadin addition reaching its maximum after 60 minutes. The differences in TJ expression between the two studies probably rely on the toxic agent administered. In fact, we used wheat gliadin instead of the peptic-tryptic (PT) digests that are known to have different modes of action in regard to their toxicity. PT treatment induces the production of alkenals that in turn can modify the activity of membrane-associated proteins and enzymes [30].

The modifications in paracellular permeability went together with a rising in the single and total polyamine content that was evident and significant after 6 h of exposure. A clear role for polyamines at cellular and molecular levels in the gliadin-triggered damage of intestinal epithelia is still under debate. Regulation of brush border functions by spermidine and spermine has been suggested to be mediated by a transglutaminase-induced incorporation of polyamines into membrane proteins [31]. Besides, it has been hypothesized that epithelial binding of gliadin peptides may occur in the form of IgA immune complexes which then translocate across the epithelium [32]. This binding could represent powerful extraneous growth factors for the gut and, as a result, induce extensive proliferation and changes in the metabolism of epithelial cells via activation of second messenger pathways. These metabolic changes may release huge amounts of polyamines, mostly spermidine [33]. On the other hand, the increase in polyamine content probably results from increased cell proliferation during the repair phase of mucosal injury. In this context, polyamine levels could be regarded as markers of a hyperproliferative state in response to toxic effects of gliadin. This behavior by polyamines has already been reported during inflammation of intestine leading to derangement of the mucosa [34].

The second aim of the study was to investigate the possible effects on paracellular permeability and polyamine content following co-administration of viable L.GG, LGG-HK or its conditioned medium with gliadin. In previous experiments by our group, L.GG was proven to be effective in modulating cell proliferation and polyamine metabolism and biosynthesis also when its components (namely cytoplasm extracts and cell wall extracts) were tested, supporting the hypothesis that intact cells is not a pre-requisite for the L.GG protective effects [19,20].

Although members of the Lactobacillus genera are a subdominant part of the normal colonic microbiota, they represent a much greater component higher up in the gut [35]. Animal models and cell culture systems have provided indications that lactobacilli are able to counteract alterations in paracellular permeability evoked by cytokines, chemicals, peptides, infections or stress [36]. A paper by Seth et al. [37] reported that the administration of live and heat inactivated L.GG, bacterial supernatants and peculiar L.GG purified soluble proteins to Caco-2 cells treated by hydrogen peroxide that destroys TER and increases permeability, caused the secretion of proteins of this strain effective against the insult.

In our study, the administration of viable and heat killed L.GG as well as its conditioned medium, caused only a slight and not significant increase in TER after 90 min from exposure without any effects on lactulose flux and zonulin release. By opposite, in Caco-2 cells treated with gliadin, the addition of viable L.GG, but also L.GG-HK and L.GG-CM, significantly restored cell barrier function. Also the single and total polyamine levels diminished significantly when Caco-2 cells were exposed to gliadin in combination with viable and heat killed L.GG. Recently, our group reported that the administration of viable, heat killed L.GG and L.GG homogenate to DLD-1 and HGC-27 cell lines significantly reduced neoplastic proliferation as well as polyamine content and biosynthesis [19,20,38].

As regards the protective effects of some probiotics against gliadin, our findings are in line with data in literature [39] and different mechanisms could be evoked to explain the effects exerted by L.GG, not only as viable bacteria, but also when they were heat inactivated or their conditioned medium was used. Firstly, L.GG might inhibit gliadin-induced damage in Caco-2 cells by hydrolyzing gliadin similarly to other live probiotic bacteria as in the VSL3# probiotic preparation [40]. These strains showed the ability to colonize the human stomach and duodenum, where the hydrolysis of gliadin epitopes may be relevant for decreasing the abnormal secretion of zonulin and the initial step of immune response to gliadin [41,42]. Secondly, the peculiar set of peptidases shown by L.GG was probably able to inhibit the gliadin-induced damage to Caco-2 cells breaking up wheat gliadin into small harmless peptide products [43]. Thirdly, L.GG might modulate directly the function of epithelial cells. It has already been reported that different probiotic strains, probiotic bacterial lysates or conditioned medium increase epithelial barrier function as measured by TER [44]. In addition, L.GG might protect the epithelium from the gliadin insult by direct action on the cells.

One interesting finding of the present study is that viable L.GG *per se* was able to significantly increase ZO-1, Claudin-1 and Occludin expression after 6 h of exposure. Even if the gliadin effects on TJ expression were significant only after 24 h, the co-administration of viable L.GG with gliadin caused an early and significant increase in the expression of the tested proteins compared to gliadin treated cells. Besides, after 24 h, viable L.GG with gliadin continued to significantly increase the expression of Claudin-1 and Occludin, but exerted only a slight and not significant decrease on ZO-1 levels. Available data support the capability of peculiar probiotic strains in modulating TJ protein expression. Pretreatment of Caco-2 monolayers with *L. plantarum* significantly attenuated the effects of phorbol ester-induced dislocation of ZO-1 and Occludin and the associated increase in epithelial permeability [45]. Additionally, treatment of Caco-2 cells with the probiotic *L. plantarum* MB452 resulted in augmented transcription of Occludin and Cingulin genes, suggesting that bacteria-induced improvements to intestinal barrier integrity may also be regulated at the gene expression level [46].

Of note, the presence of polyamines was required for viable L.GG to exert its effects on TJ expression. As a matter of fact, when Caco-2 monolayers were deprived in the polyamine content by DFMO, the expression of TJ proteins was not significantly different from that in controls or cells treated with gliadin alone. Cellular polyamines spermidine, spermine and their precursor putrescine, have been indicated as playing a role in the maintenance of the intestinal epithelial integrity by their ability to modulate expression and functions of various genes, such as intercellular junction proteins [12]. Present findings let us hypothesize that the action of viable L.GG in modulating the expression of TJ proteins could be mediated also by the presence of cellular polyamines, although the exact mechanisms are still not completely elucidated. Possibly, they may be related to the specific molecular structure of these compounds. At physiological pH, putrescine, spermidine, and spermine possess two, three, and four positive charges, respectively [47]. These compounds can bind to negatively charged macromolecules such as DNA, RNA, and proteins to influence the sequence-specific DNA-, RNA- or protein-protein interactions, which alter gene transcription and translation and the stability of mRNAs and proteins.

## Conclusions

The present study demonstrates that gliadin is able to alter the intestinal paracellular permeability and to significantly increase the polyamine content in Caco-2 cells. Concomitant administration of L.GG counteracts these effects. Interestingly, the presence of cellular polyamines is a pre-requisite for this probiotic to exert its capability in restoring paracellular permeability by affecting the expression of different TJ proteins.

For CD patients, a lifetime adherence to a strict GFD treatment is difficult to follow. Thus, alternative therapies for CD are being hypothesized, including agents that reduce gluten exposure by either binding or degrading gluten in the intestinal lumen or prevent gluten uptake into the mucosa. In this perspective, probiotic strains such as L.GG, not only as viable bacteria, but also in its heat inactivated form or conditioned medium may play a role in protecting intestinal mucosa from gliadin induced damage.

The complex relationship between probiotics and polyamines as well as the role played by these amines in maintenance of intestinal epithelial integrity justify further studies. Research will be addressed to investigate the role of polyamines by evaluating not only the enzymes involved in the regulation of their production and degradation, but also considering *in vivo* study design on animal model of gluten-sensitive enteropathy [48].

## Abbreviations

TJ: Tight junction; CD: Celiac disease; IP: Intestinal permeability; GFD: Gluten free diet; ATCC 53103 L.GG: *Lactobacillus rhamnosus* GG; FBS: Fetal bovine serum; MRS: Man-Rogosa-Sharpe; PBS: Phosphate buffered saline; CM: Conditioned medium; HK: Heat killed; TER: Transepithelial resistance; HPAEC: High performance anion exchange chromatography; PAD: Pulsed amperometric detector; 1,10-DAD: 1,10-diaminodecane; PCA: Perchloride acid; qPCR: Quantitative PCR; DFMO: α-Difluoromethylornithine; RT: Reverse transcription; AUC: Area under the curve.

## Competing interests

The authors declare that they have no competing interests.

## Authors’ contributions

AO and FR contributed equally to this work. AO, FR and ML designed the research. MN, VT, AO and ML performed the experiments. FR and AO analyzed the data and wrote the paper. All authors read and approved the final manuscript.
